# Improved Precision-Cut Liver Slice Cultures for Testing Drug-Induced Liver Fibrosis

**DOI:** 10.3389/fmed.2022.862185

**Published:** 2022-03-30

**Authors:** Liza Dewyse, Vincent De Smet, Stefaan Verhulst, Nathalie Eysackers, Rastislav Kunda, Nouredin Messaoudi, Hendrik Reynaert, Leo A. van Grunsven

**Affiliations:** ^1^Department of Basic Biomedical Sciences, Liver Cell Biology Research Group, Vrije Universiteit Brussel, Brussels, Belgium; ^2^Department of Internal Medicine, Universitair Ziekenhuis Brussel, Brussels, Belgium; ^3^Department of Surgery, Universitair Ziekenhuis Brussel, Brussels, Belgium; ^4^Department of Gastroenterology and Hepatology, Universitair Ziekenhuis Brussel, Brussels, Belgium

**Keywords:** PCLS, DILI, hepatic stellate, VPA, human, mouse, *in vitro*

## Abstract

*In vitro* models of human liver disease often fail to mimic the complex 3D structures and cellular organizations found *in vivo*. Precision cut liver slices (PCLS) retain the complex physiological architecture of the native liver and therefore could be an exceptional *in vitro* liver model. However, the production of PCLS induces a spontaneous culture-induced fibrogenic reaction, limiting the application of PCLS to anti-fibrotic compounds. Our aim was to improve PCLS cultures to allow compound-induced fibrosis induction. Hepatotoxicity in PCLS cultures was analyzed by lactate dehydrogenase leakage and albumin secretion, while fibrogenesis was analyzed by qRT-PCR and western blot for hepatic stellate cell (HSC) activation markers and collagen 6 secretion by enzyme-linked immunosorbent assays (ELISA). We demonstrate that supplementation of 3 mm mouse PCLS cultures with valproate strongly reduces fibrosis and improves cell viability in our PCLS cultures for up to 5 days. Fibrogenesis can still be induced both directly and indirectly through exposure to TGFβ and the hepatotoxin acetaminophen, respectively. Finally, human PCLS cultures showed similar but less robust results. In conclusion, we optimized PCLS cultures to allow for drug-induced liver fibrosis modeling.

## Introduction

Chronic liver diseases are the major cause of progressive liver fibrosis, which accounts for two million deaths worldwide each year (1) and is expected to further increase (2). Liver fibrosis or scarring of the liver tissue, is a wound-healing response to perpetual liver injury or hepatocyte damage. The hepatic stellate cells (HSCs) are the major cellular source of extracellular matrix (ECM) deposition, independent of the etiology of the liver injury (3). Although significant advances have been made the past few years, besides causal treatments, such as anti-viral therapies or a change of lifestyle, which can prevent, slow down or reverse fibrosis progression, no efficient anti-fibrotic liver therapies are currently available in the clinic (4). One of the main obstacles hampering the development of anti-fibrotic therapies is the lack of robust *in vitro* models that appropriately represent human liver disease. Animal models are still the most popular preclinical assessment modality, however, its value in predicting human physiological response is mostly poor, resulting in approximately 80% of potential therapies failing in clinical trials (5). Furthermore, ethical concerns are increasingly stimulating the need for alternative approaches to animal testing, especially as the 3R principle – Replacement, Reduction and Refinement of animal use – has been embedded in the legislation and guidelines concerning animal testing worldwide (6).

Traditionally, *in vitro* models of liver fibrosis rely on 2D cultures of primary HSCs. However, while these *in vitro* activated HSCs partly resemble their *in vivo* counterpart, they do not fully recapitulate *in vivo* HSC activation (7–9). The possible reason for this discrepancy is the loss of micro-environmental context in these 2D cultures. Hence, the development of characterized 3D models that can reflect the many aspects of the *in vivo* liver structures is necessary. Precision-cut liver slices (PCLS) were introduced 35 years ago as an *in vitro* liver model with great potential as it possesses all needed requirements. To obtain these cultures, fresh liver tissue is processed to yield 250 μm slices with a diameter of 3–8 mm that can be cultured in regular tissue culture plates (10). As the PCLS preparation results in cut surfaces which triggers a repair and regenerative response and thus activate the HSCs within 48 h (11), PCLS have been used as a model to test anti-fibrotic drugs (12–14). Even though several researchers have tried to prolong the PCLS culture lifespan (15, 16), PCLS are mostly used during a short period of time (24–48 h). While many (anti-)fibrotics have been tested (12, 17, 18), demonstrations of drug-induced hepatocyte-damage dependent HSC activation and fibrosis are lacking for PCLS culture set-ups (10).

In this study, we describe culture adjustments that stabilize PCLS and prolongs their lifespan to at least five days in culture using valproic acid sodium salt (VPA). We show the applicability of the improved PCLS cultures for the induction of drug-induced liver injury and liver fibrosis.

## Materials and Methods

### Animals

Healthy male BALB/c mice (Charles River, Boston, MA, United States), aged 12–30 weeks, were used. Mice were maintained in controlled temperature, humidity and a light-dark cycle from 07.00 a.m. to 07.00 p.m. and allowed food and water *ad libitum*. All experimental protocols and animal experimentation ethics were carried out in accordance with the approved guidelines of the Vrije Universiteit Brussel (VUB, Belgium) and according to European Guidelines for the Care and Use of Laboratory Animals. All animal experimentation protocols were approved by the Ethical Committee of Animal Experimentation of the Vrije Universiteit Brussel (VUB, Belgium) (LA 123 02 12, projects 18-212-1; 19-212-1).

### Human Liver Tissue

Human liver tissue was obtained from hepatectomies performed by the Department of Surgery at the University Hospital of Brussels (UZ Brussel). After surgical resection, liver tissue samples from the specimen in an area unaffected by and distant from the lesion for which surgical resection was intented for. The liver tissue was immediately put in ice cold IGL-1 solution (Institute Georges Lopez). The ethical approval Reference 2015/278; B.U.N. 143201525406 was obtained from the ethical committee of the UZ Brussel and was in accordance with the Declaration of Helsinki. All participants signed their informed consent prior to the donation of liver tissue. An overview of human patient characteristics is given in Table 1.

**TABLE 1:** Patient characteristics.

Donor ID	Age	Gender	Liver disease	Surgery type
A9	56	Female	Cholangiocarcinoma (IPN-B)	Left hepatectomy
A12	85	Male	Liver metastasis (CRC)	Partial right hepatectomy
A13	55	Male	Liver metastasis (CRC)	Right hepatectomy
A14	76	Female	Liver metastasis (CRC)	Partial left hepatectomy
A16	68	Male	Primary hilar cholangiocarcinoma	Left hepatectomy
A39	69	Female	Hepatocellular carcinoma	Central hepatectomy
A40	66	Female	Polycystic liver disease	Left hepatectomy
A42	70	Male	Hepatocellular carcinoma	Partial right hepatectomy

*Overview of human patient characteristics used for human PCLS cultures. IPB-B, intraductal papillary neoplasm of the bile duct; CRC, colorectal cancer.*

### Production and Culture of Precision-Cut Liver Slices

Mice were anesthetized using an i.p. injection with 100 μl Dolethal^®^ (Vetoquinol). After anesthesia of the mouse, the median liver lobe was isolated and immediately put on ice cold IGL-1 solution. Liver tissue was sliced using a Leica VT1200S vibrating blade microtome (Leica Biosystems; speed: 0.1 mm/s, amplitude: 2 mm, step size: 250 μm), while being submerged in Williams’ Medium E medium (Gibco), supplemented with or without 2.5 mM VPA (Sigma-Aldrich). Next, the 250 μm thick sections were subsequently punched with a disposable 3 mm biopsy puncher with plunger (Kai Medical). We analyzed PCLS with different diameters (1, 3, or 8 mm) for liver culture stability. The RNA yield of discs with 1 mm diameter was not sufficient for robust and reproducible quantitative real-time polymerase chain reaction (qPCR) analysis. 3 mm diameter discs, compared to 8 mm diameter discs, resulted in a better maintenance of mRNA levels of hepatocyte markers for at least 3 days (Supplementary Figure 1). Using a P1000 pipet with cut tip, PCLS were transferred into a 24-well plate, pre-filled with cold supplemented WME medium. After slicing and punching was finished, PCLS were refreshed with pre-heated WME medium (Gibco), supplemented with 1% Ultraglutamine I (Lonza) and 1% Penicillin-Streptomycin (Life Technologies) and cultured on an orbital shaker (Infors Celltron) at a speed of 80 rpm. PCLS were cultured at 37°C, and refreshed 2–4 h after production and every 24 h.

### PCLS Exposures

For exposures to compounds, PCLS were cultured in 2.5 mM VPA until day 3 of culture. From day 3 to day 5 of culture, VPA concentration was reduced to 1 mM and PCLS were simultaneously exposed to compounds or corresponding solvent control. Compounds concentrations used: 10 ng/mL TGF-β1 (PreproTech), 1 mM acetaminophen (APAP) (Sigma-Aldrich), 10 μM SB-525334 (Alk5 inhibitor) (Sigma-Aldrich), 20 mM N-acetyl-L-cysteine (NAC) (Sigma-Aldrich). On day 5 of culture (after a 48 h exposure), PCLS and culture medium were collected for analysis.

### Lactate Dehydrogenase Analysis

For viability analysis, PCLS culture medium was collected and centrifuged (8 min, 640 g). Supernatant was transferred to a new tube and diluted 1/5 in lactate dehydrogenase (LDH) storage buffer (200 mM Tris–HCl, 10% glycerol, 1% BSA) and stored at −80°C until analysis. LDH levels were measured using the LDH-Glo Cytotoxicity Assay (Promega) according to manufacturer’s instructions.

### mRNA Analysis

Slices were snap frozen in liquid nitrogen and stored at −80°C until RNA extraction. Total RNA was extracted with TRIzol Reagent (Thermofisher) according to the manufacturer’s manual. Samples were homogenized by crushing the tissue with 5 mm steel beads (Qiagen) with a Retsch MM 400 laboratory mill at 30 Hz for 2 min. Afterward, RNA was reverse transcribed into cDNA using the MLV Reverse Transcriptase (Promega). For qPCR, GoTaq qPCR Master Mix with BRYTE green (Promega) was used. qPCR was done using the Quantstudio3 real-time PCR system (Thermofisher). Gene-specific primers (Table 2) were produced by Integrated DNA Technologies (IDT Leuven). For analysis according to the Delta–Delta threshold (Ct) method, each Ct value was normalized against the respective reference genes (for mouse: the mean of *Gapdh* and *Gtf2b*; for human: the mean of *GAPDH and YWHAZ*). The best reference genes were selected out of six genes using GeNORM, performed in R environment. Each data point corresponds to one PCLS.

**TABLE 2:** Primer list.

Primer name	Forward primer	Reverse primer	Refseq
*Gapdh*	CCTGCTTCACCACCTTCTTG	TGTCCGTCGTGGATCTGAC	NM_008084
*Gtf2b*	ATTGGCAAGGGTACAGGAGC	GAGGTTGATTCTGTCCGCCA	NM_145546.1
*Collagen1a1*	GCTCCTCTTAGGGGCCACT	CCACGTCTCACCATTGGGG	NM_007742
*Collagen5a2*	GAAAGGCTGGTGATCAAGGT	CTTTCTCCCCGAGGTCCTAA	NM_007737.2
*Acta2*	CCAGCACCATGAAGATCAAG	TGGAAGGTAGACAGCGAAGC	NM_007392
*Lox*	TGTACGCTGTGACATTCGCT	CACTGGGAACTGGGCTTCTT	NM_010728
*Pdgfrb*	TGCAGAGACCTCAAAAGGTG	CCTGATCTTCCTCCCAGAAA	NM_001146268.1
*Albumin*	TTCTCCTTCACACCATCAAGC	ATGAGATTCTGACCCAGTGTTG	NM_009654.4
*Cyp3a11*	TGAATATGAAACTTGCTCTCACTAAAA	CCTTGTCTGCTTAATTTCAGAGGT	NM_007818.3
*GAPDH*	AGCCACATCGCTCAGACAC	GCCCAATACGACCAAATCC	NM_002046.4
*YWHAZ*	ACTTGACATTGTGGACATCGGA	GTGGGACAGCATGGATGACA	NM_001135699.1
*COL1A1*	CCGGCTCCTGCTCCTCTTAGCG	CGTTCTGTACGCAGGTGATTGGTGG	NM_000088.3
*ACTA2*	CTGTTCCAGCCATCCTTCAT	TCATGATGCTGTTGTAGGTGG	NM_001141945.2
*LOXL2*	GGAGAGGACATACAATACCAAAGTG	CCATGGAGAATGGCCAGTAG	NM_002318.2

*Exon spanning primers were designed to analyze gene expression using real time PCR.*

### miRNA Analysis

PCLS culture medium was collected and centrifuged (8 min, 640 g) and stored at −80°C until analysis. For the analysis, 500 μl of medium was used. miRNA was extracted using the Nucleospin^®^ miRNA Plasma kit (Macherey-Nagel) according to the manufacturer’s protocol. *Caenorhabditis elegans* miRNA-39 (cel-miR-39) (Qiagen) was spiked into the lysate before extraction and served as an external processing control.

### Western Blot

Snap frozen PCLS were dissolved in RIPA lysis buffer (1% NP-40, 0.1% sodium-deoxycholate, 0.04% SDS, 50 mM Tris–HCl, 10 mM NaF, 30 mM NaCl, and 0.4mM EDTA), supplemented with complete protease-inhibitors (Roche Diagnostics) and PhosSTOP phosphatase inhibitors (Roche Diagnostics). Lysates were sonicated twice (15 s, 50% amplitude) in a 4°C water bath using a digital sonifier (Branson). Protein concentrations were quantified using the Micro BCA™ Protein assay kit (Thermo Fisher Scientific) according to manufacturer’s instructions. Thirty micrograms of total protein was used for western blotting. Protein expression was assessed using antibodies against β-actin (1:5,000, Sigma-Aldrich), PDGFRβ (1:1,000, Abcam), Albumin (1:1,000, Novus Biologicals), and αSMA (1:5,000, Vendor, Lifespan Technologies). Protein bands were visualized with an ImageQuant™ LAS 4000 (GE Healthcare Life Sciences). Protein bands were quantified using ImageJ (NIH).

### Enzyme-Linked Immunosorbent Assays

PCLS culture medium was centrifuged (8 min, 640 g). Supernatant was transferred to a new tube and stored at −80°C until use. Secreted Albumin or Collagen 6 protein levels were measured with a commercially available ELISA kit (MyBiosource, Albumin: MBS2516177, Collagen 6: MBS2704928). Protocol was executed according to the manufacturer’s instructions. Absorbance values were obtained with an iMark™ microplate absorbance reader (Bio-Rad).

### Hematoxylin-Eosin Staining

PCLS were fixed for 10 min in formalin and stored in PBS at 4°C until embedding. Paraffin embedded PCLS were sliced in 4 μm sections. Next, sections were deparaffinized and rehydrated. After rehydration, sections were incubated for 3 min in hematoxylin (Carl Roth), followed by a rinse with tap water and acidified water. Next, sections were rinsed in running tap water for 10 min, followed by a 3 min incubation in eosin (Sigma-Aldrich) staining. After the staining, the sections were shortly rinsed with water and subsequently dehydrated through graded washes of ethanol and water. Sections were mounted in DPX mounting medium.

### RNA Sequencing and Downstream Analysis

After RNA extraction, RNA was processed for RNASeq by NovaSeq SP 100 × 6 bp single-end sequencing. This resulted in, on average, 7 M reads per sample. Quality control and trimming was performed using FastQC and STAR was used for mapping of the reads to the reference genome (Mus_musculus_GRCm38.p6) (19). The python package StringTie was used for assembly of genes and transcripts (20). After generation of raw counts, DESeq2 in R was used to normalize counts, determine differentially expressed genes and perform principal component analysis (PCA) (21). The Database for Annotation, Visualization, and Integrated Discovery (DAVID) v6.8 was used to perform Gene Ontology (GO) analysis (22). GSEA software v4.1.0 was used to perform gene set enrichment analysis (GSEA) (23). For GSEA analyzes, gene set permutation type and a false discovery rate (FDR) statistic threshold of 0.1 was chosen to determine significantly enriched gene sets. Bulk RNAseq data of day 3 PCLS cultures has been deposited in the GEO public data base under accession number GSE194128.

### Schemes

All schemes were created with Biorender.com using an Academic License.

### Statistical Analysis

Data was analyzed using Graphpad Prism 8 (Graphpad, Palo Alto) statistical software. As our data is considered being non-parametric, quantitative variables are expressed as boxplots (median – min to max). In case of two conditions, a Mann–Whitney test was performed, or in case of more than two conditions, Kruskal–Wallis test with Dunn’s *post hoc* test were performed. For each graph, *n* = x indicates how many biological repeats were performed, with one biological repeat being one different mouse/human liver.

## Results

### Standard PCLS Cultures Do Not Allow Drug-Induced Liver Injury and Fibrosis Modeling

**FIGURE 1:**
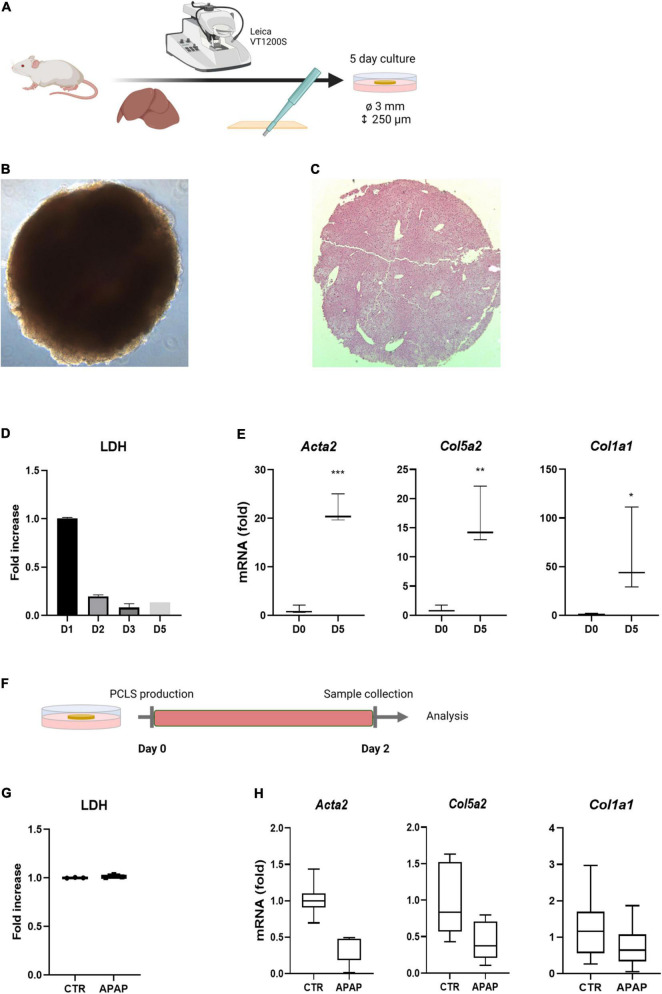
Standard culture conditions do not allow drug-induced fibrosis modeling. **(A)** Liver tissue is sliced with a vibratome and subsequently punched with a biopsy puncher, resulting in 250 μm thick discs with a 3 mm diameter. PCLS are cultured for 5 days in regular WME medium. **(B)** Microscopic picture of freshly sliced 3 mm liver slice. **(C)** H&E staining of a freshly sliced 3 mm liver slice. **(D)** Medium of cultured PCLS shows high levels of LDH leakage on day 1, which are strongly reduced from day 2 on (*n* = 3). **(E)** mRNA levels of HSC activation markers is significantly increased upon culture time. Fold increase to day 0 (*n* = 3). **(F)** PCLS were exposed to 1 mM APAP the first 48 h of culture and medium and slices were analyzed for respectively. **(G)** LDH levels (*n* = 3) **(H)** mRNA levels of HSC activation markers. Fold increase to untreated 48 h PCLS cultures (*n* = 6). *p ≤ 0.05, **p ≤ 0.01, ***p ≤ 0.001.

PCLS cultures have frequently been used for compound metabolization and toxicity testing. Several studies have evaluated the anti-fibrotic effects of drugs and direct activation of HSCs by TGFβ or/and PDGF-BB can be easily induced (16). However, the evaluation of drug-induced liver fibrosis and HSC activation has not been well documented yet (10). We set out to evaluate whether it is possible to induce HSC activation in traditional PCLS cultures by exposing them to acetaminophen (APAP), shown to transiently induce HSC activation after an acute exposure (24), and fibrosis when chronically administered to mice (25). PCLS with 3 mm diameter and 250 μm thickness were obtained by slicing mouse liver tissue with a Leica VT1200S vibrating blade microtome. Subsequently, liver discs were punched with a 3 mm core biopsy puncher and cultured for 5 days in regular WME medium (Figure 1A). Figure 1B shows a freshly sliced 3 mm disc imaged using light microscopy and Figure 1C a 4 μm hematoxylin-eosin (H&E) section of the disc showing the intact liver architecture of the disc. The production and culture of PCLS induces a cut surface, which results in cell death. When PCLS were cultured for 5 days in regular WME medium, high levels of LDH, representing cell death, were detected on day 1 of culture which strongly reduced from day 2 onward (Figure 1D). This hepatocyte damage eventually results in a fibrotic response over culture time as demonstrated by the significant increase in mRNA levels of genes associated with HSC activation (*Acta2, Col5a2*, and *Col1a1*) on day 5 of culture (Figure 1E). Exposure of PCLS to 1 mM acetaminophen (APAP) during the first 48 h of culture (Figure 1F) demonstrated that an (additional) hepatocyte-damage and subsequently induced pro-fibrotic response could not be established (Figures 1G,H). This suggests that in standard PCLS conditions, the injury associated with the production of the slices is most likely too high and additional damage by drugs, and subsequent fibrosis, is not easily achieved.

### Valproic Acid Improves PCLS Culture Conditions and Represses HSC Activation

**FIGURE 2:**
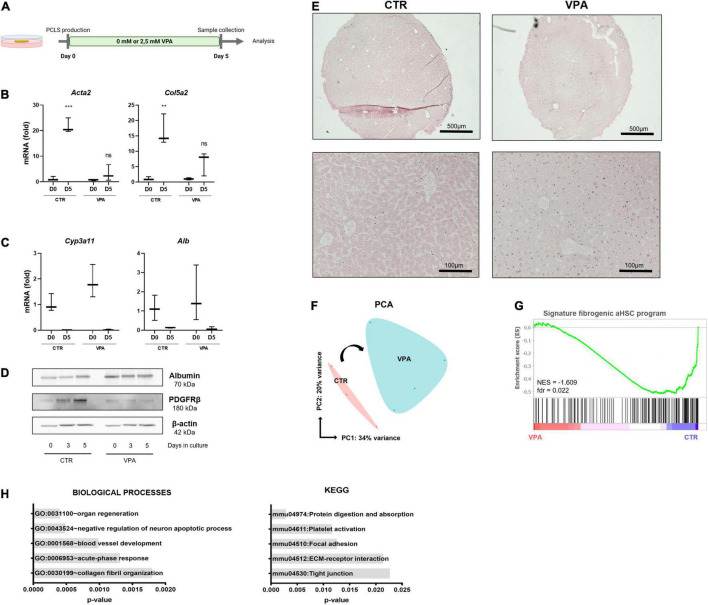
Valproic acid improves PCLS culture conditions and represses the pro-fibrotic signature. **(A)** PCLS were cultured in the absence (control) or presence of VPA (2.5 mM VPA) for 5 days. **(B)** VPA supplementation reduces HSC activation (*n* = 3) but **(C)** does not affect hepatocyte marker mRNA levels (*n* = 3). **(D)** PDGFRβ protein expression was reduced, and albumin protein expression was slightly higher over time in the presence of VPA. **(E)** H&E staining on day 5 shows better preservation of liver histology when PCLS cultures are VPA supplemented. **(F)** PCA plot of PCLS cultures with/without VPA shows division of both conditions over PC1 (*n* = 4). **(G)** GSEA analysis shows that the fibrogenic aHSC gene signature is significantly enriched in the standard culture conditions but not in VPA supplemented cultures. **(H)** GO analysis of biological processes (left) and KEGG analysis (right) of downregulated genes in PCLS cultured with VPA. **p ≤ 0.01, ***p ≤ 0.001.

VPA, a histone deacetylase class I inhibitor, is known to inhibit HSC transdifferentiation and activation (26). We hypothesized that if we could inhibit the first wave of HSC activation, which is induced during the initial damage to hepatocytes as a result of the slicing and punching procedure, we would perhaps be able to induce drug-induced liver damage and fibrosis at a later time point of the cultures. To investigate this, PCLS were cultured for 5 days with regular WME or medium supplemented with 2.5 mM VPA (Figure 2A), the dose that can inhibit culture-induced activation of primary mouse HSCs (26). The addition of 2.5 mM VPA to the culture medium had beneficial effects on the procedure-induced fibrosis progression, as HSC activation genes were not significantly upregulated after 5 days of culture in the presence of VPA, in contrast to the regular standard conditions (Figure 2B). This tendency was also observed on protein level, as PDGFRβ protein expression did not increase over time in the presence of VPA (Figure 2D). Hepatocyte-specific gene expression was not different on day 5 when comparing both conditions (Figure 2C). However, albumin protein expression remained slightly higher over time in the presence of VPA (Figure 2D). Moreover, H&E staining shows a better preservation of cell nuclei and liver histology and architecture on day 5 when PCLS were cultured in the presence of 2.5 mM VPA (Figure 2E).

To get more insight into the underlying mechanisms of the beneficial effects of VPA for the cultures, RNA sequencing (RNA-seq) of PCLS cultured for 3 days in the absence (CTR) or presence (VPA) of 2.5 mM VPA was performed. PCA plot shows a division of CTR and VPA treated slices over PC1, which accounts for 34% of variance (Figure 2F). Moreover, the fibrogenic aHSC gene signature recently identified by De Smet et al. (9), is significantly enriched in standard PCLS culture conditions when compared to VPA supplemented conditions (Figure 2G). Differential gene expression analysis showed that 48 genes were differentially upregulated and 130 genes were differentially downregulated (such as *Aebp1, Col1a1*, and *Col3a1)* in the presence of VPA compared to control conditions (data not shown). Further downstream analysis demonstrates that VPA downregulated genes are associated with the GO category “ECM organization” and “collagen fibril organization” and the KEGG pathway “ECM-receptor interaction” (Figure 2H). This data demonstrates that VPA extends the life span of PCLS cultures up to 5 days as tissue integrity is better preserved and the procedure-induced fibrogenic aHSC transcriptional program is strongly repressed.

### VPA Supplemented PCLS Cultures Allow *in vitro* Modeling of Drug-Induced Liver Injury and Fibrosis

**FIGURE 3:**
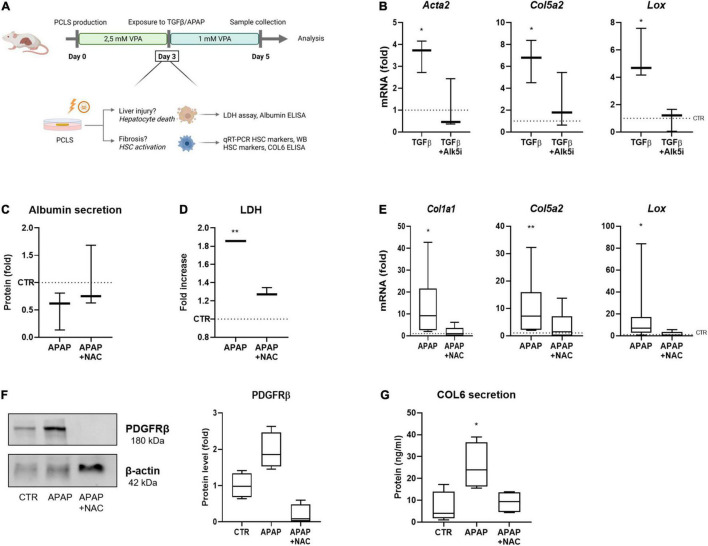
PCLS for *in vitro* modeling of drug-induced liver injury and fibrosis. **(A)** General overview of the improved conditions of our mouse PCLS cultures. PCLS are cultured in 2.5 mM VPA between day 0–day 3. From day 3 until day 5, VPA concentration is reduced to 1 mM VPA, while PCLS are exposed to TGFβ or APAP. PCLS are collected on day 5 and are analyzed for liver injury or fibrosis. **(B)** Exposure of PCLS to TGFβ induces an increased RNA expression of HSC activation markers (*n* = 3). **(C)** PCLS exposed to APAP show a decreased albumin secretion (*n* = 3), **(D)** an increase in LDH leakage (*n* = 3), **(E)** increased RNA expression of HSC activation markers (*n* = 9), **(F)** increased protein expression of HSC marker PDGFRβ (*n* = 4), and **(G)** an increased secretion of collagen 6 in the culture medium (*n* = 4). The dotted line represents the corresponding control. *p ≤ 0.05, **p ≤ 0.01.

Next, we wanted to use the VPA-supplemented conditions to test for drug-induced liver injury and fibrosis induction. However, we noticed that in VPA supplemented PCLS cultures induction of HSC activation with TGFβ at day 3 was not very robust, and that complete wash-out of VPA from day 3 until day 5 induced HSC activation genes (Supplementary Figure 2A). However, reduction of the VPA concentrations to 1 mM from day 3 to day 5, did not result in a significant induction of HSC activation (Supplementary Figure 2B). We used this modified setup, further referred to as PCLS^V^, to test direct HSC activation by TGFβ during 48 h starting at day 3 (Figure 3A). Exposure of these PCLS^V^ cultures to TGFβ at day 3 clearly induced HSC activation as evidenced by an increase in mRNA levels of HSC activation markers *Col5a2, Acta2*, and *Lox* (Figure 3B). TGFβ-induced HSC activation could be inhibited by co-exposing PCLS^V^ with SB-525334 (Alk5i), a selective inhibitor of TGFβRI (27). As this experiment demonstrated that the HSCs were still functional at that time point, i.e., able to respond to a direct stimulus, we next investigated whether HSC activation (liver fibrosis) could be induced to the same extent by damaging the hepatocytes with APAP (28). Exposure to APAP induced hepatotoxicity, as APAP administration slightly reduced albumin secretion in the culture medium (Figure 3C), and resulted in a clear increase in LDH leakage (Figure 3D).

To confirm whether this increased LDH leakage was due to the cytotoxic effect of APAP, PCLS^V^ cultures were treated with NAC, which is currently still the only antidote used in the clinic for APAP-induced liver injury (29). NAC is known to block APAP-induced hepatotoxicity, as it acts as a substitute of glutathione, when administered within 8 h after overdose in patients (30). However, studies have shown that NAC treatment in mice is not effective beyond 4 h after APAP administration, as murine have an accelerated metabolism (31). Therefore, NAC was administered simultaneously with APAP to the PCLS^V^ cultures at day 3. Indeed, the co-administration of APAP with NAC prevented the decreased secretion of albumin (Figure 3C) and decreased the LDH leakage (Figure 3D). The APAP- induced hepatocyte damage resulted in a pro-fibrotic response as demonstrated by an increase in *Col1a1, Col5a2*, and *Lox* expression after APAP administration, while this effect was blocked when NAC was co-administered (Figure 3E). The same tendencies were observed at the protein level, as PDFRβ expression in the slices (Figure 3F) and collagen 6 secretion in the culture medium (Figure 3G) were increased in APAP exposed PCLS^V^, while this was not observed in the presence of NAC. These results show that in the PCLS^V^ cultures, the hepatotoxic effect of APAP results in a pro-fibrotic response of the HSCs, which can be inhibited through NAC administration.

### Human PCLS Cultures Allow Hepatic Stellate Cell Activation

**FIGURE 4:**
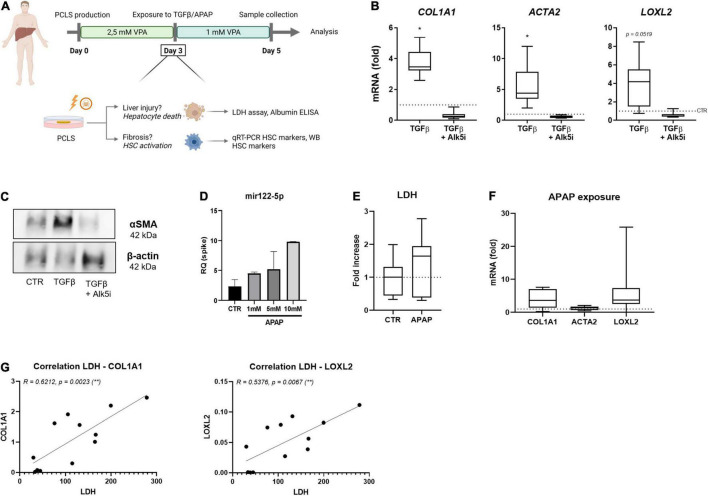
Human PCLS^V^ cultures allow HSC activation modeling. **(A)** Human PCLS were cultured for 3 days in the presence of 2.5 mM VPA. On day 3 of culture, PCLS were exposed to 10 ng/ml TGFβ or 1 mM APAP, while VPA concentration was reduced to 1 mM for the last 48 h. On day 5 of culture, PCLS were analyzed. **(B)** mRNA levels of HSC activation markers were analyzed by qRT-PCR after 48 h exposure to TGFβ in the absence or presence of its inhibitor Alk5i (*n* = 9). **(C)** Western blot analysis of αSMA protein expression in the human PCLSV culture is increased upon TGFβ exposure. **(D)** miRNA122-5p expression in culture medium of human PCLSV exposed to different concentrations of APAP (*n* = 3). **(E)** LDH levels in culture medium of human PCLSV exposed to 1 mM APAP for 48 h (*n* = 7). **(F)** mRNA expression of HSC activation markers *COL1A1*, *ACTA2* and *LOXL2* in PCLS^V^ exposed to 1 mM APAP for 48 h (*n* = 7). **(G)** The correlation of LDH leakage and HSC activation markers COL1A1 and LOXL2 of untreated human PCLS^V^ on day 5 of culture. *p ≤ 0.05, **p ≤ 0.01.

Lastly, we investigated whether our findings obtained in mouse PCLS^V^ cultures could be extrapolated to human PCLS cultures. Therefore, human PCLS^V^ cultures were established and tested for their fibrotic response at day 3 following TGFβ- or APAP exposure (Figure 4A). Human PCLS^V^ cultures exposed to TGFβ show increased expression of HSC activation markers on mRNA (Figure 4B) and protein level (Figure 4C). This effect could be very efficiently blocked by the inhibitor SB-525334 (Alk5i).

Human PCLS^V^ exposed to increasing concentrations of APAP did however not result in an increase of LDH leakage (Figure 4E). However, a dose-dependent increase of mir122-5p, a miRNA that can be used as a surrogate of hepatocyte damage (32), was detected in human PCLS^V^ culture medium (Figure 4D). Unfortunately, although there was a clear trend for the induction of *COL1A1* and *LOXL2* mRNA levels, due to the large variations in the extend of activation, these increases were not significant (Figure 4F). These large variations in HSC activation are most likely due to the great difference in health status of our donor livers, as a strong correlation was found between LDH leakage and mRNA HSC activation levels on day 5 of culture (Figure 4G).

## Discussion

PCLS cultures are a very useful tool in metabolism studies and the testing of anti-fibrotic drugs (12, 17, 18), however drug-induced liver injury dependent HSC activation and fibrosis is poorly documented in PCLS cultures (10). In this study, we aimed at establishing culture conditions that would allow the induction of HSC activation depending on drug-induced liver damage instead of only depending on the damage of the slices that is intrinsic to the PCLS procedure. We now show that supplementation of the standard WME culture conditions with 2.5 mM VPA results in a better preservation of liver histology, a strong reduction in HSC activation and an improved conservation of the hepatocyte metabolic activity, as the hepatocytes remain capable of processing APAP. In PCLS^V^ cultures, HSCs do not only remain more quiescent, but are also still able to activate to a direct trigger such as TGFβ and to indirect triggers such as an APAP-induced hepatocyte damage (Figure 3). Finally we showed that these PCLS^V^ conditions can be used for human PCLS cultures but does not result in such a robust induction of HSCs when they are exposed to APAP.

Most PCLS studies test anti-fibrotic compounds as the procedure (slicing) induces a tissue repair- and regenerative response, which in its turn results in fibrosis (11). Although direct stimulation of HSC using TGFβ and/or PDGF-BB results in a robust pro-fibrotic response in PCLS cultures (16), a hepatocyte-damage dependent HSC activation is less characterized in PCLS cultures. Several studies show the (hepatocyte) toxicity of the exposure of e.g., APAP (33, 34) or CCl_4_ (35) in PCLS cultures. Vatakuti et al. used APAP administration, along with other compounds to induce necrosis or cholestasis in human PCLS cultured with the aim to classify cholestatic and necrotic hepatotoxicity. While they clearly showed that PCLS are a useful model to predict the phenotype of drug-induced hepatotoxicity, fibrosis or HSC activation were not documented (33). Granitzny et al. extensively characterized the APAP-induced toxicity in rat PCLS in flasks, but no downstream analysis for fibrosis or HSC activation was carried out (34). CCl_4_ has been used to demonstrate fibrosis induction in PCLS by two studies. Van de Bovenkamp et al. (36), administered 5 μl CCl_4_ to rat PCLS in 25 ml flasks and documented an increase of α*B-crystalin* and *Klf6* mRNA expression after 16 h of exposure, but no fibrosis induction by collagen levels was shown. Ten years later, Sadasivan et al. showed that CCl_4_ addition in the first 24 h of mouse PCLS cultures (150 μm thickness) in 25 ml flasks, did not induce extra hepatotoxicity at that time point, but it did affect mRNA levels of *Acta2* and *Col1a1* (35). The PCLS^V^ conditions presented here allows the induction of fibrosis in a controlled way, which we demonstrated by using the known hepatotoxic agent APAP in regular 24 well plates. We believe that these conditions not only allow the detection of pro-fibrotic compounds that possibly could not exceed the basal “fibrosis threshold” in regular culture conditions, but could also be used to screen for anti-fibrotic drugs. Although upscaling of the currently used PCLS^V^ 24-well plate setup to 48 wells could facilitate small scale screening of compounds, the current PCLS^V^ conditions are difficult to translate to a high throughput set-up since the diameter of the slices is 3 mm.

In this study, APAP was used as a model compound for the induction of liver fibrosis at a concentration of 1 mM. This concentration has been proven to be clinically relevant as it has been shown that plasma peak concentrations of APAP of 1–2 mM induce hepatotoxicity in patients (37). However, differences in species and sex should be kept in mind. For instance, in this study we have made use of male BALB/c mice while female mice are less sensitive to APAP-induced liver damage, as female mice restore their GSH levels more efficiently (38). Moreover, a majority of PCLS research studies make use of rats to produce PCLS. However, literature has shown a limited relevance of rats for APAP studies, as this species has been proven to be less susceptible to APAP, and develops only minor liver injury compared to mice, even when exposed to high doses (39).

Not only the choice of species (40) or sex, but also the experimental design could possibly affect study outcome. In this study PCLS with a diameter of 3 mm were used, whereas the majority of PCLS research papers – according to the protocol of De Graaf et al. (41) – describe the use of 5 or 8 mm discs (10). So far, no study has compared the use of different PCLS sizes. In our experimental setup, we observed a better preservation of hepatocyte functionality in smaller size discs (Supplementary Figure 1), but a more thorough analysis comparing different diameters and different thickness of slices for mouse, rat, and human could determine which condition would be best to use in future experiments.

The PCLS^V^ conditions were applied to human PCLS cultures to investigate whether we could use the optimized conditions for human slices as well. In such human PCLS^V^ cultures we can easily induce direct HSC activation by TGFβ exposures but drug-induced hepatocyte damage was more difficult to obtain. This is most likely due to the high variability, and already poor health status of the donor livers that we were able to use. As our human donors carry different disease etiologies and are of different ages, substantial differences in basal HSC activation levels and LDH leakage levels were observed (Figure 4G). Moreover, three patients with colorectal cancer (CRC) underwent chemotherapy pre-operation, which is known to be hepatotoxic (42) and may have affected our observed results as well. Similar to previous findings (43), this might be the underlying reason why we were not able to induce a consistent drug-induced fibrotic response.

## Conclusion

In conclusion, through supplementation of the standard PCLS culture medium with VPA we established PCLS^V^ cultures that allow the modeling of drug-induced HSC activation and fibrosis. This opens up possibilities for compound screenings for targets of drug-induced liver fibrosis and also offers possibilities to model other chronic liver disease, such as cholestasis or non-alcoholic fatty liver disease (NAFLD).

## Data Availability Statement

The datasets presented in this study can be found in online repositories. The names of the repository/repositories and accession number(s) can be found below: https://www.ncbi.nlm.nih.gov/geo/, GSE194128.

## Ethics Statement

The studies involving human participants were reviewed and approved by the Ethical Committee of the UZ Brussel. The patients/participants provided their written informed consent to participate in this study. The animal study was reviewed and approved by the Ethical Committee of Animal Experimentation of the Vrije Universiteit Brussel (VUB, Belgium).

## Author Contributions

LD: conceptualization, investigation, methodology, formal analysis, validation, visualization, data curation, and writing. VD: formal analysis. SV: formal analysis. NE: methodology. RK and NM: provision human patient tissue and formal analysis. HR: supervision and funding acquisition. LG: supervision, conceptualization, funding acquisition, data curation, and writing. All authors contributed to the article and approved the submitted version.

## Conflict of Interest

The authors declare that the research was conducted in the absence of any commercial or financial relationships that could be construed as a potential conflict of interest.

## Publisher’s Note

All claims expressed in this article are solely those of the authors and do not necessarily represent those of their affiliated organizations, or those of the publisher, the editors and the reviewers. Any product that may be evaluated in this article, or claim that may be made by its manufacturer, is not guaranteed or endorsed by the publisher.
