# Supplementary material for: Improved Precision-Cut Liver Slice Cultures for Testing Drug-Induced Liver Fibrosis
Source: Front Med (Lausanne). 2022 Mar 30;9:862185. doi: 10.3389/fmed.2022.862185 (PMC9007724; doi:10.3389/fmed.2022.862185)
Supplement: Supplementary file 1 [file Data_Sheet_1.docx]

Improved precision-cut liver slice cultures for testing drug-induced liver fibrosis

Dewyse Liza^1^, De Smet Vincent^1^, Verhulst Stefaan^1^, Eysackers Nathalie^1^, Kunda Rastislav^2^, Messaoudi Nouredin^2^, Reynaert Hendrik^1,3^, van Grunsven Leo A.^1*^

^1^Department of Basic Biomedical Sciences, Liver Cell Biology research group, Vrije Universiteit Brussel, Brussels, Belgium

^2^Department of Surgery, Universitair Ziekenhuis Brussel, Brussels, Belgium

^3^Department of Gastroenterology and Hepatology, Universitair Ziekenhuis Brussel, Brussels, Belgium

Supplementary Material


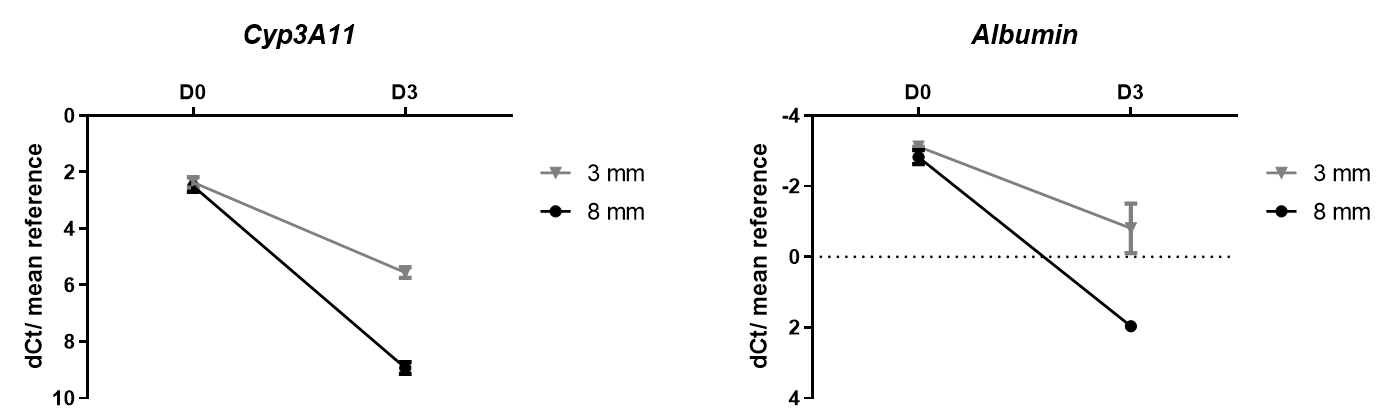


Supplementary Figure 1: Stability of hepatocyte markers in different size PCLS cultures. Gene expression of hepatocyte markers in 3 or 8 mm discs during 3 days of culture.


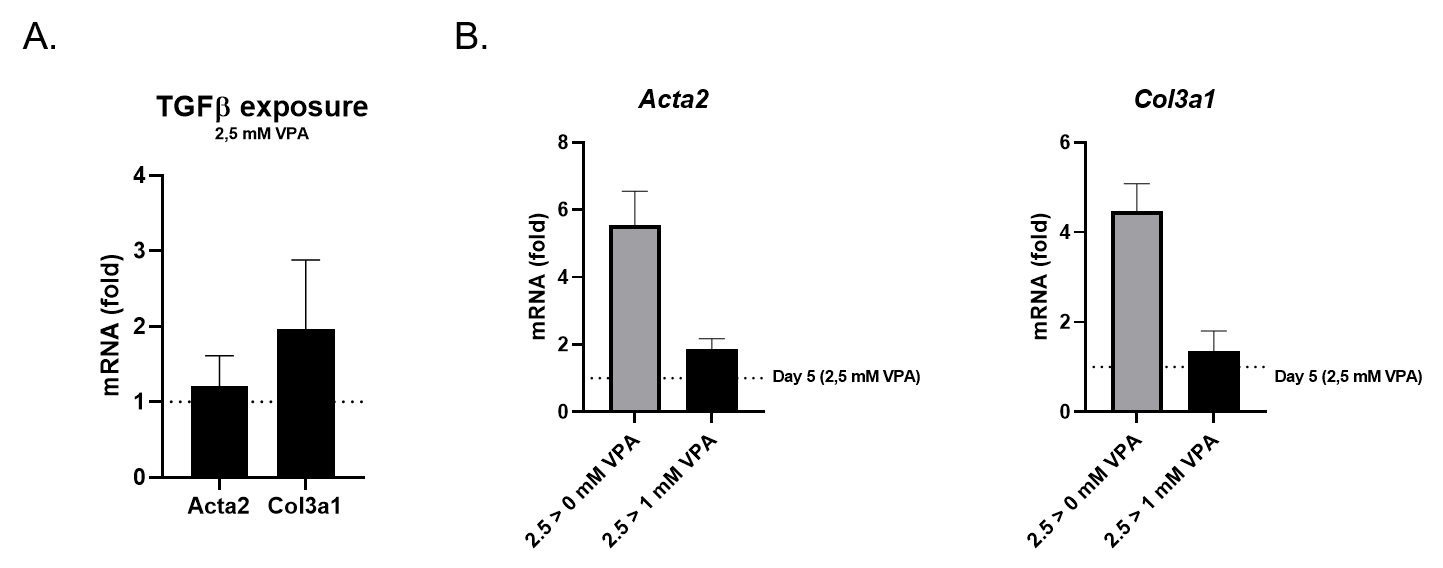


Supplementary Figure 2: VPA experiments. A) Activation markers were analyzed in PCLS exposed to TGFβ for 48 hours in the presence of 2,5 mM VPA (from day 3-5). B) mRNA levels of HSC activation markers Acta2 and Col3a1 in PCLS, cultured in 2,5 mM VPA until day 5 or when VPA concentration was reduced to 0 mM or 1 mM VPA from day 3 to day 5.
